# Insights into the Role of Tick Salivary Protease Inhibitors during Ectoparasite–Host Crosstalk

**DOI:** 10.3390/ijms22020892

**Published:** 2021-01-17

**Authors:** Mohamed Amine Jmel, Hajer Aounallah, Chaima Bensaoud, Imen Mekki, Jindřich Chmelař, Fernanda Faria, Youmna M’ghirbi, Michalis Kotsyfakis

**Affiliations:** 1 Laboratory of Genomics and Proteomics of Disease Vectors, Biology Centre CAS, Institute of Parasitology, Branišovská 1160/31, 37005 České Budějovice, Czech Republic; amine.jmel@paru.cas.cz (M.A.J.); chayma.bensaoud@paru.cas.cz (C.B.); imen.makki@paru.cas.cz (I.M.); 2 Institut Pasteur de Tunis, Université de Tunis El Manar, LR19IPTX, Service d’Entomologie Médicale, Tunis 1002, Tunisia; aounallahhajer@gmail.com (H.A.); youmna.mghirbi@pasteur.tn (Y.M.); 3 Innovation and Development Laboratory, Innovation and Development Center, Instituto Butantan, São Paulo 05503-900, Brazil; fernanda.faria@butantan.gov.br; 4 Faculty of Science, University of South Bohemia in České Budějovice, 37005 České Budějovice, Czech Republic; chmelar@prf.jcu.cz

**Keywords:** protease inhibitors, proteases, tick saliva, drug discovery

## Abstract

Protease inhibitors (PIs) are ubiquitous regulatory proteins present in all kingdoms. They play crucial tasks in controlling biological processes directed by proteases which, if not tightly regulated, can damage the host organism. PIs can be classified according to their targeted proteases or their mechanism of action. The functions of many PIs have now been characterized and are showing clinical relevance for the treatment of human diseases such as arthritis, hepatitis, cancer, AIDS, and cardiovascular diseases, amongst others. Other PIs have potential use in agriculture as insecticides, anti-fungal, and antibacterial agents. PIs from tick salivary glands are special due to their pharmacological properties and their high specificity, selectivity, and affinity to their target proteases at the tick–host interface. In this review, we discuss the structure and function of PIs in general and those PI superfamilies abundant in tick salivary glands to illustrate their possible practical applications. In doing so, we describe tick salivary PIs that are showing promise as drug candidates, highlighting the most promising ones tested in vivo and which are now progressing to preclinical and clinical trials.

## 1. Introduction

Proteases are ubiquitous enzymes in plants, animals, and microorganisms that play key roles in the majority of physiological processes [1,2]. Proteases are involved in several reversible and irreversible reaction cascades including hormone production/signaling pathways, apoptosis, inflammatory reactions, and homeostasis [3]. Depending on the active amino acid in the enzyme’s active site, proteases are classified into cysteine, serine, aspartic, glutamic, and threonine proteases [3], and metalloproteases represent another protease class containing a divalent metal ion linked to the active site residues [4]. Serine proteases are the most abundant proteolytic enzymes, followed by metallo- and cysteine proteases and finally the aspartic and threonine proteases [5]. In addition to their roles in vital biochemical processes, proteases are also implicated in various diseases such as viral diseases, cancer, inflammation, and bleeding disorders [6]. Given their important roles in diverse physiological processes, protease activity must be rigorously controlled and regulated to avoid any enzyme dysregulation that might be pathogenic to the host organism [7]. This tight regulation is usually conducted by blocking the zymogen, the inactive enzyme precursor, or through the action of protease inhibitors (PIs) [8], which partially or totally inhibit enzymes by forming a complex with their target proteases [4].

While the majority of PIs are proteins or peptides, some low molecular weight non-proteinaceous compounds such as polysaccharides, glycerolipids, triterpenes, and polyphenols are also considered PIs [9]; these are not specific to particular proteases and inhibit a broad spectrum of enzymes [10]. In contrast, proteinaceous PIs are usually more specific and can even target unique proteases. As a result, PIs can be classified according to their target enzyme, although exceptions are frequently encountered, such as the α2-macroglobulin that inhibits proteases of different classes [6]. A particularly impressive role for PIs has been observed in parasite–host crosstalk; PIs found in tick saliva have been shown to modulate host immune cells [11], mediating local immunosuppression and modulating blood clotting at the site of infection, thereby exerting a beneficial effect to the tick (allowing attachment and feeding) at the expense of the host [12].

Given their fundamental roles and potential translational application, there have been significant efforts to identify new PIs from various sources and study those already identified in detail using novel technologies and methods. Indeed, with technological advances, the study of PIs has substantially improved over recent years, not least the availability of three-dimensional (3D) structural information for several PIs and their targeted proteases, permitting receptor-based design.

Here, we review the biochemistry and fundamental mechanisms of action of PIs. We enumerate and discuss the different classes of PIs based on the proteases they inhibit and their mechanism of action. Moreover, we discuss their applications in critical fields like agriculture and medicine. In the final section, we focus on an interesting natural source of PIs, tick salivary glands, and their potential pharmacological applications.

## 2. Classification of Protease Inhibitors

The catalytic activity of proteases is regulated by different inhibition mechanisms and different PI families [13]. Despite similarities in the 3D structure of PIs, they can be classified into over 107 families and divided into 40 clans according to their structural similarities (secondary and tertiary) and their different functions [13]. Laskowski and Kato first developed a classification scheme for PIs in 1980 [14] according to their reactive center, disulfide bond number, and amino acid sequence [14]. With advances in biotechnology and increasing knowledge about PIs, Rawlings et al. [15] established a classification of PIs in 2004 based on amino acid sequence homology that subclassified PIs to 48 families and 26 clans [5].

### 2.1. Target-Based Classification

**Figure 1:**
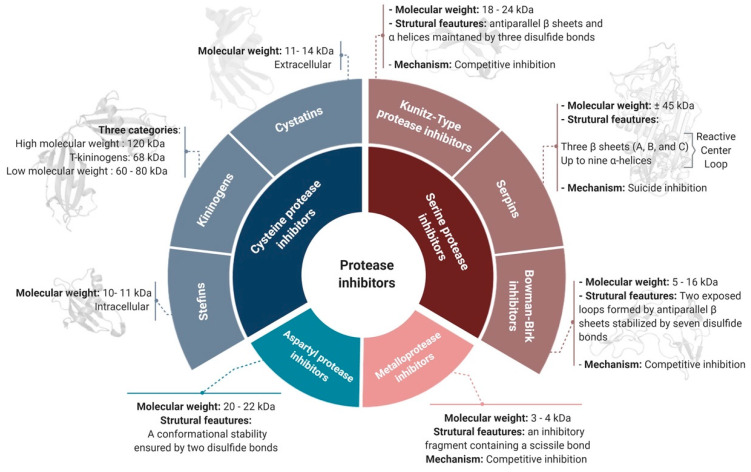
The target-based classification of protease inhibitors.

PIs can be classified according to their target protease into six groups [4]: serine, cysteine, aspartyl, glutamate, and threonine protease inhibitors. Metalloprotease inhibitors are also contained within this classification, as they inhibit proteases with a divalent metal ion in their active site [16]. A non-exhaustive list of the most common PI families, their principal features, and their properties according to the target-based classification system is illustrated in Figure 1 and discussed below.

#### 2.1.1. Serine Protease Inhibitors

Since serine proteases are the most abundant protease family, containing >26,000 proteases [17], their respective inhibitors are the largest group of PIs in animals, plants, and microorganisms [18]. Most serine protease inhibitors follow the conventional mechanism of inhibition through the generation of irreversible Michaelis complexes characterized by covalent bonds between the protease and the inhibitor [19]. Serine PI domains ensure efficient functionality and allow their subclassification into various superfamilies such as the Kunitz-type PIs, Bowman–Birk inhibitors, serpins, trypsin inhibitor-like domain inhibitors (TILs), and Kazal domain inhibitors [12].

##### Serpins

The serpin superfamily is the largest serine PI family [20]. Serpins typically weigh ~45 kDa and are relatively large molecules (~350–400 amino acids) compared to other PIs [21]. The 3D structure of serpins includes three β-sheets (A, B, and C) and up to nine α-helices that fold to form a specific spatial structure including a reactive center loop (RCL) near the C-terminus [22]. Serpins are categorized as “suicide inhibitors” due to the inactive covalent complex formed with their target protease [23]. This suicide inhibition is often referred to as a “mousetrap”, since the RCL interacts with the target protease active site, and its scissile bond (P1-P1’) is cleaved to generate a stable complex [24]. The resulting bond between the protease and the RCL leads to the insertion of the cleaved RCL into the β-sheet A and relocation of the protease to the opposite pole of the serpin, forming a suicide covalent complex [24]. Serpins are involved in the regulation of different physiological processes such as blood coagulation, fibrinolysis, signal transduction, the complement cascade, and immune responses [25].

##### Kunitz-Type PIs

PIs in the Kunitz superfamily are characterized by the presence of one or many Kunitz inhibitory domains. They are generally small proteins with molecular weights ranging from 18 to 24 kDa [26]. The Kunitz domain is characterized by anti-parallel β-sheets and α-helices maintained in a compact 3D structure by three disulfide bonds [27]. Most Kunitz-type PIs are competitive inhibitors, acting in a substrate-like manner and binding reversibly to the protease [28]. Active site blocking is mediated by the RCL attachment to the catalytic zone through a non-covalent bond. The highly exposed RCL loop of Kunitz-domain inhibitors is suitable for a wide variety of proteases, so these inhibitors are relatively non-specific and therefore potentially useful across a range of applications [29]. Indeed, Kunitz-type PIs are known to regulate inflammation and coagulation factors and have also been implicated in tumor biology [3].

##### Bowman–Birk Inhibitors (BBIs)

This superfamily of PIs is characterized by small molecular weight peptides ranging from 5 to 16 kDa and a structure with a single or two inhibitory regions [30]. BBIs are competitive inhibitors and follow the classical mechanism of substrate binding to the protease active site [31]. A single BBI inhibitor protein can act on two different target proteases simultaneously by virtue of two opposed loops formed by antiparallel β-sheets and stabilized by seven disulfide bonds [23,32]. Given their specific mechanism, several researchers have focused on BBIs for specific applications such as to inhibit cancer [33].

#### 2.1.2. Cysteine Protease Inhibitors

Cysteine protease inhibitors (CPIs), or cystatins, are the second largest group of PIs after serine PIs. They are divided into three main families: family-1 cystatins or stefins, family-2 cystatins or cystatins, and family-3 cystatins or kininogens. Stefins are mostly intracellular and the smallest cystatin family in terms of molecular weight (10–11 kDa) [3]. Stefins inhibit cathepsins B, L, and H and also papain. In several therapeutic investigations, they have been identified as potential diagnostic tools for cancer [34]. Like stefins, cystatins inhibit papain and cathepsins B, L, and H, but, they are larger (11–14 kDa) and are transported out of the cell to exert their action [35]. Kininogens are divided into three categories: high molecular weight kininogens (120 kDa), T-kininogens (68 kDa), and low molecular weight kininogens (60–80 kDa) [3,36,37]. They play important roles in the modulation of inflammatory responses and are used as biomarkers of kidney disorders [37].

There are numerous documented functions of CPIs, and some have been shown to be critical for the proper functioning of important physiological pathways such as cathepsin regulation [38]. The structural features of CPIs include 4 to 5 antiparallel β-sheets surrounding an α-helix. Their highly conserved inhibitory domain is mainly composed of two hairpin-like loops formed by the β-sheets and the N-terminal region [39]. Cystatins follow the competitive inhibition model with slight modifications, as they do not bind in a substrate-like manner. In fact, the two hairpin loops bind to the protease active site and block the access of any substrate, while the N-terminal region maintains effective attachment of the inhibitor to the enzyme [40,41].

#### 2.1.3. Metalloproteases Inhibitors

Despite their low molecular weight (only 3–4 kDa), metalloprotease inhibitors (MPIs) effectively inhibit a wide range of metalloproteases [42]. MPIs are classified as competitive inhibitors, since they act in a substrate-like manner [43]. From a structural point of view, MPIs do not possess an inhibitory loop or a specific secondary structure for inhibition. Instead, their inhibitory fragment is located near the C-terminus and contains a scissile bond. The cleavage of this latter bond allows the fixation of the new C-terminal side to the active site of the protease with the help of its metallic ion [23,28]. The efficiency of MPI inhibition has been reported to be enhanced by secondary interactions outside the active site of the protease [44].

#### 2.1.4. Aspartyl Protease Inhibitors

The natural aspartyl PIs are proteins of about 20 to 22 kDa and conformational stability ensured by two disulfide bonds [45]. Despite aspartyl proteases including important members such as cathepsins D and E, renin, pepsin A and C, and, most importantly, the HIV-1 protease, their natural inhibitors remain poorly described for several reasons. One main reason is probably related to the low representation of aspartyl proteases in the human genome, with only 15 members described [46]. Regardless of their low bioavailability, the presence of a scissile bond and their short half-life have meant that strategies to inhibit aspartyl proteases involve the development of synthetic peptides or mimics with a non-cleavable bond to replace the scissile bond [47].

### 2.2. Mechanism-Based Classification

**Figure 2:**
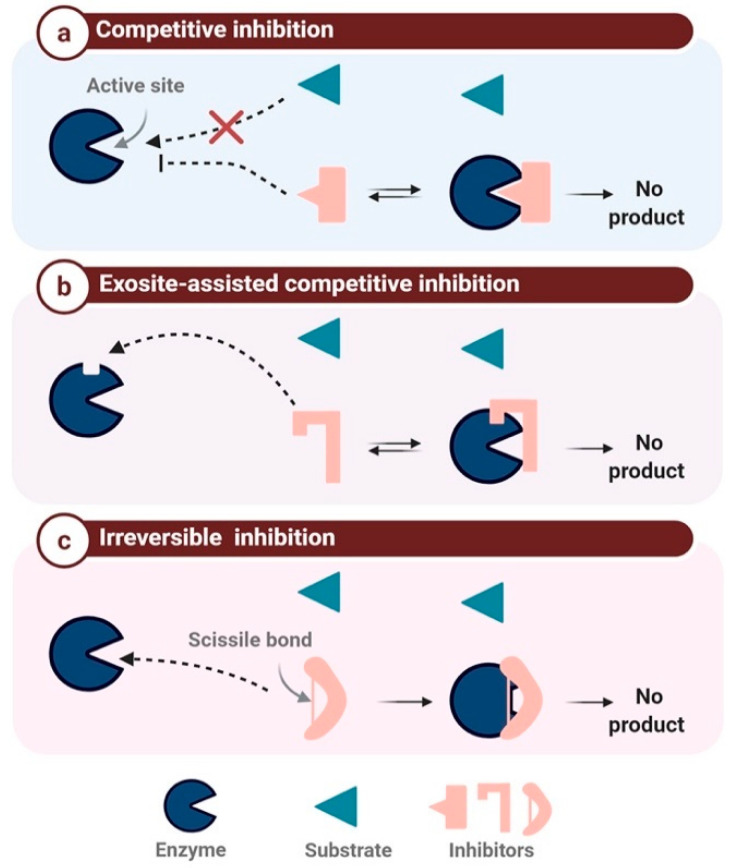
The mechanism-based classification of protease inhibitors. (**a**): competitive inhibition: the inhibitor binds to the active site instead of the substrate, (**b**): Exosite-assisted competitive inhibition: the inhibitor blocks the access to the active site through binding to an exosite, (**c**): Irreversible inhibition: the inhibitor binds irreversibly to the enzyme inducing its inactivation

The target-based classification is limited, as numerous PIs are active against two or more enzymes. Indeed, in humans, there are vastly more proteases than PIs; despite the continuous discovery of human proteases and their respective inhibitors, the ratio of one PI for every five proteases has remained constant [41,48]. However, in addition to the target-based classification, it is possible to classify PIs according to their mechanism of inhibition, in particular the steric or catalytic inhibition of the enzyme active site or its neighboring regions [49]. Enzyme inhibition mechanisms can be divided into two general categories, reversible and irreversible. Reversible inhibition can be further subdivided into competitive, uncompetitive, and non-competitive inhibition [41]. The mechanism based-classification can be divided into three major classes (Figure 2): competitive protease inhibition (also called canonical inhibition), exosite-assisted competitive inhibition or non-canonical inhibition, and finally irreversible inhibition or trapping inhibition [41].

#### 2.2.1. Competitive Protease Inhibitors

The canonical inhibition mechanism is also known as the standard or Laskowski mechanism [50]. Inhibitors belonging to this class attach using a lock-and-key system through the insertion of the inhibitor RCL into the catalytic site of the targeted protease (Figure 2a). The β-sheet conformation allows the binding of the inhibitor to the active site in a substrate-like manner. Consequently, the RCL scissile bond is slowly hydrolyzed by the protease without any product release, as the amide bond is later reconnected [15,41]. Numerous PIs belong to this family, mainly the BBIs and Kazal and Kunitz domain-containing inhibitors.

#### 2.2.2. Exosite-Assisted Competitive Inhibitors

Known also as non-canonical inhibitors, this class represents inhibitors binding to a secondary site distinct from the protease active site (Figure 2b). Access to this active site is blocked in a non-catalytic manner [51,52]. This inhibition mechanism is classified as competitive but differs from the standard Laskowski mechanism [50]. Inhibitor binding to the exosite is crucial for inhibition, as it maintains the inhibitor–enzyme interaction and enhances inhibitor specificity [51]. As mentioned above, CPIs are non-canonical PIs that follow a slightly modified competitive inhibition mechanism, since the N-terminal region does not interact with the active site in a substrate-like manner but switches to the side of the active site to ensure sufficient binding energy for the enzyme–inhibitor complex [41,53].

#### 2.2.3. Irreversible Inhibition

This class of inhibition is triggered by the protease, which catalyzes the activation of its respective inhibitor (also referred to as a suicide substrate). The cleavage of the inhibitor reactive loops (Figure 2c) triggers a major conformational change, resulting in the irreversible cross-linking of the protease to its inhibitor [23,51]. α-2-macroglobulin is a 600 kDa inhibitor with four reactive loops on its surface, which plays a major role in the elimination of excessive proteases in the blood [54]. Serpins are also well-known suicide inhibitors, as described above [55].

## 3. Applications of Protease Inhibitors

The accelerating increase in PI-related knowledge (structure, mechanism of action, functional applications) has paved the way for several biotechnological applications of PIs related to medicine and agriculture, as summarized in Table 1.

Biomedical research efforts have underscored the major role of these inhibitors in pathobiology, providing opportunities to exploit them for the treatment of diseases such as HIV [56] and cancer [57]. PIs targeting human proteases are implicated in protease-related blood coagulation disorders, cancers, immune system dysfunction, and neurodegenerative diseases [4]. In HIV infections, several combinations of PIs have been used therapeutically [58], and protease inhibitor-based drugs such as nucleoside/nucleotide reverse transcriptase inhibitors (NRTIs) or non-nucleoside reverse transcriptase inhibitors (NNRTIs) have been developed as antiretroviral drugs [59]. In cancer, PIs have been tested as antitumor therapies to target the proteases implicated in tumor progression and metastasis such as matrix metalloproteases, cathepsin B, matriptase, and kallikreins [60]; for example, a BBI caused apoptosis and cell cycle arrest in colon cancer (HT-29) and breast cancer (MCF-7) cell lines [61]. Moreover, a BBI mixture from soybean was shown to have a cancer chemo-preventive effect against neoplastic polyps and was applied to the preclinical detection of prostate cancer [36]. Additionally, proteases are common in the immune system and neurodegenerative diseases [32]. Several interesting properties, such as their high target specificity, have made PIs interesting candidates for drug discovery [2]. Over 70 PIs have been commercialized as drugs, with over 150 examined in clinical trials and thousands preclinically [23]. Among the commonly-used commercialized PI drugs, enalapril and captopril inhibit angiotensin-converting enzyme and are used to treat cardiovascular disease, and bivalirudin is a thrombin inhibitor and potent anticoagulant [62,63].

In agriculture, plant PIs have been thoroughly investigated as they are important in plant defense mechanisms. With the increased use of genetic engineering, transgenic plants with genes coding for PIs have been investigated to improve agriculture. Indeed, the PI dose applied to plants was correlated with protection against phytopathogens, insects, microbes, and pests [64,65]. As a result, PIs have been employed in agriculture as insecticides [66], anti-fungal agents [67], and antibacterial agents [68]. They target the digestive proteases of phytophagous entities to exert anti-proliferative and growth inhibitory effects [69]. Transcriptomic studies have revealed that PIs are the main proteins produced during plant exposure to external pathogens [70]. A previous in silico study of soybean plants revealed that a multigene family coding for Bowman–Birk PIs contained 11 BBI genes that were expressed in a synchronized manner and that played a crucial role in host-plant defenses against pathogens [71,72]. However, further studies of plant PIs are required to overcome persistent challenges such as the adaptation of some insects by downregulating their targeted proteases and upregulating other proteases (e.g., gut proteases) to avoid the neutralization of their enzymes [73].

PIs are regulated after saturation by available or free inhibitors, and other reports have indicated that they are inactivated through oxidation of their active sites or through proteolysis catalyzed by specific non-complexing proteases [94]. CPIs, for example, can be degraded by neutrophil elastase, which is an extracellular serine protease [95]. On the other hand, PI expression and synthesis are mainly stimulated by the presence of inactive inhibitors or by an increase in PI complexes [96].

## 4. Tick Salivary Glands: An Attractive Source of PIs with Potential Pharmacological Applications

**Figure 3:**
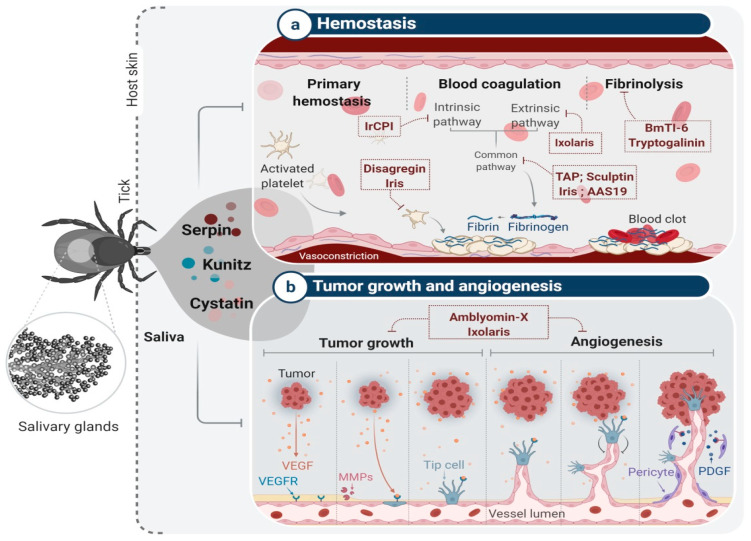
Examples of protease inhibitors from tick salivary glands with anti-hemostatic, anti-tumor and anti-angiogenic effects. (**a**) Numerous PIs from tick salivary glands have been characterized as anti-hemostatic compounds targeting platelet aggregation such as Disagregin and Iris, blood coagulation including IrCPI, Ixolaris, TAP, Sculptin, Iris, and AAS19, and fibrinolyses like BmTI-6 and Tryptogalinin. (**b**) With great interest, Amblyomin-X and Ixolaris were found to significantly inhibit tumor growth with efficient anti-angiogenic action.

Ticks are obligate hematophagous arthropods that transmit the greatest variety of pathogenic microorganisms to a wide range of wild and domestic animals, as well as humans [97]. Hard ticks (Ixodidae) are unique among blood-feeders, lacerating small vessels and sucking blood from the resulting hematoma for several days or even weeks [97]. Meanwhile, the host develops various responses to minimize blood loss and to reject the tick including hemostasis, innate and acquired immunity, and inflammatory responses, leading to wound healing and tissue remodeling [98]. Most of these responses rely on proteolytic pathways involving several proteases, in particular pro-coagulants (thrombin, coagulation factors), pro-inflammatory enzymes (neutrophil elastase, proteinase 3, chymase, tryptase, kallikrein, and cathepsins L, B, S, C, and G, etc.), and complement enzymes [99,100,101]. To complete their blood meal, ticks must hijack the host defense mechanisms triggered by the tick bite and the accompanying infection [98]. To do so, they secrete a wide variety of pharmaco-active molecules into the host via their saliva to modulate the proteolytic balance in the bite site to guarantee an interrupted blood meal [102]. These molecules include PIs with anti-clotting, anti-platelet, anti-tumor and anti-angiogenic effects, and that interfere with defense-related host proteases (Figure 3) [103].

**Figure 4:**
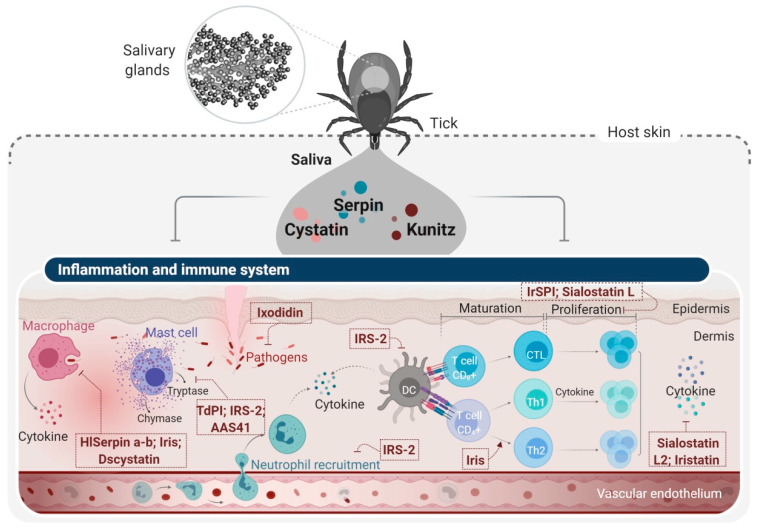
Examples of protease inhibitors from tick salivary glands with immunomodulatory effects. Several PIs from tick salivary glands have been described for their anti-inflammatory activity such as HlSerpin a-b, Dscystatin, Ixodidin, TdPI, and AAS41 or as immunomodulatory candidates including Iris, IRS-2, Sialostatin L2, Iristatin, and IrSPI.

Other PIs were rigorously described for their immunomodulatory and anti-inflammatory effects targeting both innate and adaptive host immunity (Figure 4) [103]. Apart from their role in sustaining tick feeding, PIs represent the most prominent protein families in tick saliva that promote the establishment of tick-borne pathogens in the host [104]. Other tick PIs target proteases involved in tick digestion, mainly those degrading hemoglobin and albumin [105], or proteases implicated in embryogenesis that degrade reserve proteins and energy supplies during the early phases of the tick lifecycle [106]. There are a few studies reporting tick salivary PIs that facilitate successful pathogen transmission to hosts or that have a role in tick digestion and embryogenesis and are reviewed elsewhere [103].

PIs from tick salivary glands have long aroused interest in scientists in the field due to their pharmacological characteristics and their high specificity, selectivity, and affinity to their target proteases in the host [12]. As a result, research into tick PIs has steadily progressed. Primarily, PIs have been purified from tick saliva or salivary gland extracts and then characterized in several biochemical assays [107]. Nevertheless, some downstream assays require milligrams or even grams of purified protein, and obtaining this amount of native protein from crude tick saliva fractions or salivary gland extracts is challenging and any purified proteins are likely to be contaminated [108]. However, the development of new protein production methods using diverse expression systems has been a milestone in drug discovery, in particular using the highly cost-effective *Escherichia coli* expression system [109,110,111]. With advances in high-throughput approaches such as transcriptomics and proteomics, several tick PI-encoding cDNAs have been cloned, and individual recombinant PIs have been thoroughly studied [12]. Indeed, the expansion of sialoma projects (from the Greek sialo = saliva) over the three last decades has enabled the annotation of thousands of tick salivary sequences that are attributable to different protein families and are now available in public databases [112]. These projects have unraveled two abundant PI families in tick salivary glands: serine PIs and cysteine PIs [12]. Specifically, four superfamilies of serine PIs have been identified in tick saliva including kunitz domain-containing inhibitors, serpins, trypsin inhibitor-type cysteine-rich domain inhibitors (TIL domain inhibitors), and kazal domain inhibitors, while cysteine PIs refer to the cystatin family [12]. Several PIs, principally kunitz-type inhibitors, serpins, and cystatins, have now been rigorously characterized in vitro and in vivo [102,113,114], but only a limited number have progressed to pre-clinical and clinical trials due to strict criteria for clinical use [11].

### 4.1. Tick Serine Protease Inhibitors and Their Applications

Kunitz domain inhibitors are highly abundant in tick salivary secretions and are usually associated with the inhibition of trypsin-like serine proteases [115]. As mentioned above, members of this family can contain multiple tandem repeats of Kunitz domains [115]. In tick salivary glands, only proteins with a single (monolaris), two (bilaris), or five (penthalaris) Kunitz inhibitory domains have been detected [116]. Proteins from this superfamily are considered to be the most valuable serine PIs in tick salivary glands given their wide range of possible applications.

Tick salivary Kunitz domain inhibitors mostly function as anti-thrombotic factors by inhibiting various proteases in the coagulation cascade and involved in platelet aggregation [117]. Most of these inhibitors target thrombin, factor Xa, factor XIIa, trypsin, and elastase [117]. For instance, Ir-CPI [118] and TAP [119] were reported as anticoagulants, and Disagregin was identified as a platelet aggregation inhibitor. Ir-CPI is a multi-target Kunitz-type inhibitor that specifically interacts with activated human contact phase factors (FXIIa, FXIa, and kallikrein), thereby inhibiting the intrinsic coagulation pathway [118]. The administration of Ir-CPI in both rat and mouse venous and arterial thrombosis models resulted in a significant dose-dependent reduction in venous thrombus formation and revealed a defect in the formation of arterial occlusive thrombi [118]. In addition, mice injected with Ir-CPI were protected against collagen- and epinephrine-induced thromboembolism without bleeding or impaired blood coagulation parameters [118]. Recently, the antithrombotic activity of Ir-CPI was evaluated in preclinical animal models, confirming that Ir-CPI is an effective and safe antithrombotic agent [120]. TAP, on the other hand, is a single target Kunitz-domain anticoagulant peptide from *Ornithodoros moubata* saliva [119] first identified as a slow tight-binding competitive inhibitor of FXa [119]. The recombinant form (rTAP) was shown to significantly inhibit thromboplastin-induced fibrinopeptide A generation following infusion into conscious rhesus monkeys [119]. TAP has been tested in diverse models of venous and arterial thrombosis. In a primate model of arterial thrombosis, rTAP demonstrated an antithrombotic effect and inhibited blood clotting more effectively than heparin, a commonly used anticoagulant in patients with cardiovascular disease, acute thrombosis, and arrhythmias [121,122]. In another study, TAP-antibody had antithrombotic effects at low doses in a murine ferric chloride-induced carotid artery thrombosis model [123]. Despite its promising anti-thrombotic effects, TAP has yet to be tested in humans, mainly due to its antigenicity. However, both studies hypothesized that direct FXa inhibitors could be used as an effective pharmacological approach for the prevention of high-shear arterial thrombosis and other diseases such as atherosclerosis or atrial fibrillation. In contrast to TAP, Disagregin, also isolated from *O. moubata*, is a potent Kunitz-type inhibitor and selective inhibitor of fibrinogen-dependent platelet aggregation and the adhesion of platelets to fibrinogen [124]. Disagregin inhibits platelet aggregation in plasma stimulated by several agonists such as ADP, thrombin, platelet-activating factor, and collagen [125]. Apart from having a unique sequence, the authors of this study demonstrated that Disagregin binds to GPIIb-IIIa through a mechanism distinct from that used by RGD-containing disintegrins [125]. These anti-hemostatic Kunitz-type compounds may be useful therapeutically to treat other cardiovascular or hematological diseases.

In addition to their anti-hemostatic proprieties, some tick PIs have been described as having anti-tumor and anti-angiogenic therapeutic properties, such as Ixolaris and Amblyomin-X [126,127]. Ixolaris is a pleiotropic two-Kunitz domain PI isolated from the salivary glands of *Ixodes scapularis*, with promising antithrombotic [126], anti-angiogenic, and antitumor proprieties [128]. Recombinant Ixolaris was expressed in insect cells and shown to behave as a fast-and-tight ligand of FXa exosites, thereby inhibiting factor VIIa (FVIIa)/tissue factor (TF)-induced factor X (FX) activation [128,129,130]

A recent structural study revealed that Ixolaris interacts with FXa via a noncanonical mechanism [131]. Ixolaris also reduces heparin-catalyzed inhibition by antithrombin III by binding to plasmatic FX and weakens the binding of FXa to plasmatic or immobilized heparin [129,132]. In its antithrombotic activity, Ixolaris caused a dose-dependent reduction in thrombus formation in a venous thrombosis model by inhibiting the extrinsic pathway of the coagulation cascade [133]. Due to its potent and long-lasting activity with no apparent hemorrhage or bleeding side-effects, Ixolaris is considered to be an effective and possibly safe antithrombotic agent [133]. Given the mutual relationship between cancer and its blood supply [134], several studies have investigated the effect of Ixolaris on tumor growth and tumor vascularization. First, Ixolaris has been shown to inhibit the growth of U87-MG cells in nude mice without visible bleeding [135], and the inhibitory effect of Ixolaris on tumor growth was accompanied by downregulation of vascular endothelial growth factor (VEGF) and a decrease in tumor vasculature in a human glioblastoma model [135,136]. In recent preclinical studies, Ixolaris administered in a murine melanoma model retained inhibitory activity on blood coagulation and showed a significant anti-metastatic effect compared to untreated controls [126]. Taken together, these findings suggest that Ixolaris might be a promising anticancer and antiangiogenesis therapeutic, especially for metastatic melanoma.

Amblyomin-X is a well-studied Kunitz-type inhibitor from tick salivary glands that has undergone advanced pre-clinical testing. Amblyomin-X is a non-competitive inhibitor of FXa with a unique structure that acts on prothrombinase and tenase complexes. It was identified in the salivary glands of *Amblyomma cajennense* (currently *A. sculptum*) [137] and was first described as an anti-coagulant [137,138] and later intensively investigated for its anti-tumor and anti-angiogenic effects [139,140]. Although the effects of Amblyomin-X on blood coagulation might be relevant to its antitumor effects, it has also been shown to have direct, non-hemostatic effects on cells such as proteasome and autophagy inhibition [134]. Amblyomin-X selectively acts on tumor cells and induces apoptotic cell death, inhibiting murine melanoma growth in vivo and decreasing the number of metastatic events [141]. Moreover, Amblyomin-X induced apoptosis in murine renal cell carcinoma in a dose-dependent manner, provoking mitochondrial dysfunction and stimulating the production of reactive oxygen species (ROS) [139]. Apart from its anti-tumor effects, Amblyomin-X inhibits VEGF-A-induced angiogenesis by modulating endothelial cell proliferation and adhesion in the chicken chorioallantoic membrane (CAM) [142,143]. Moreover, Amblyomin-X modulates Rho-GTPases and uPAR signaling and reduces the release of MMPs, thereby disrupting the actin cytoskeleton and decreasing the cellular migration of tumor cell lines [141]. In a pre-clinical study, Amblyomin-X was administered to mice harboring orthotopic kidney tumors and significantly decreased lung metastasis [144]. Impressively, the injected dose of Amblyomin-X was safe, with any symptoms of toxicity being reversible and only seen at higher doses. In a horse melanoma model, Amblyomin-X modulated the tumor immune microenvironment by inducing tumor cell death and significantly reducing the tumor size [144].

Other Kunitz-type proteins have been described as immunomodulatory compounds, particularly anti-inflammatory candidates. From *I. ricinus*, IrSPI, a Kunitz elastase inhibitor, exhibited immunomodulatory activity by repressing the proliferation of CD4^+^ T lymphocytes and pro-inflammatory cytokine secretion from both splenocytes and macrophages [145]. BmTI-6 is a Kunitz-type trypsin and plasmin inhibitor isolated from *Rhipicephalus (Boophilus) microplus*, which attenuated the pathophysiological and inflammatory parameters of induced emphysema in mice [146]. Kunitz domain-containing proteins were also found to inhibit tryptases released from mast cells during the inflammatory process. Tick-derived protease inhibitor (TdPI) from the salivary glands of *R. appendiculatus* is a tight-binding Kunitz-related inhibitor of human tryptase β [147]. TdPI was also found to suppress the activity of trypsin and plasmin, but with lower specificity. In one model, TdPI entered mouse mast cells, accumulated in their cytosolic granules, and prevented the autocatalytic activation of tryptase, thereby suppressing inflammation [147]. Tryptogalinin from *I. scapularis*, in addition to inhibiting β-tryptase, also targeted other serine proteases such as α-chymotrypsin, plasmin, matriptase, and elastase involved in inflammation and tissue remodeling [148]. Both TdPI and Tryptogalinin are therefore promising candidates for the treatment of allergic inflammatory disorders like asthma [147,148,149].

Aside from inhibiting proteases, proteins with this domain can also target ion channels [150,151]. It has been shown that a modified Kunitz domain peptide from the salivary glands of *R. appendiculatus* can activate potassium channels in an in vitro system, suggesting a vasodilator function [152]. These properties have made Kunitz domain inhibitors attractive potential new remedies targeting various life-threatening diseases [134].

Serpins are the largest family of serine protease inhibitors, present in all kingdoms and the second most abundant PI superfamily in tick salivary glands [153]. Like their mammalian counterparts, tick serpins appear to execute their function through a suicide cleavage mechanism in which both the serpin and the targeted protease are permanently inactivated after recognition [153]. Of note, some tick serpins can inhibit multiple enzymes including AAmS6, AAS27, and AAS41 from *A. americanum* [154,155,156], IxscS-1E1 from *I. scapularis* [157], and RmS-6 and RmS-17 from *R. microplus* [158]. Rather than being considered promiscuous, they appear to be selective in the sense that the targeted enzymes are often part of a conserved biological mechanism. One well-studied protein to illustrate this point is AAS19 from the salivary glands of *A. americanum*, which inhibits blood clotting factors Xa, IXa, XIIa and XIa, thrombin, trypsin, and plasmin, all of which are involved in the hemostatic system [159,160].

Investigations into several tick serpins have provided a fundamental understanding of the molecular basis of their roles in tick biology, reproduction, parasitism, and blood feeding [153]. From this perspective, various possible applications of tick salivary serpins have been proposed based on extensive in vitro and in vivo studies. Nevertheless, to our knowledge, none of these serpins has reached preclinical trials, possibly due to their high molecular weight. As serpins are relatively “big” molecules, typically ~45 kDa but up to 100 kDa due to differences in their glycosylation profiles, this could be problematic when designing new drugs [161]. Encouragingly, a recent study investigated the activity of the SA-RCL peptide derived from the RCL domain of the tick serpin HlSerpin-a, which displayed similar enzymatic inhibitory activity and immunosuppressive properties to full-length HlSerpin-a [162]. By virtue of their proprieties, various applications of tick salivary serpins have emerged. For instance, RHS8 from the tick *R. haemaphysaloides* was studied to understand tick reproduction, given its role in vitellogenesis [163]. rSerpin from *R. microplus* [164], ***Iris*** from *I. ricinus* [165], and AAS19 from *A. americanum* have been proposed as candidate anti-tick vaccines. The immunization of rabbits and/or mice with recombinant rSerpin and Iris increased the mortality of feeding ticks and reduced their weight after engorgement [165]. The vaccination of rabbits with rAAS19 resulted in faster feeding, smaller ingested blood volumes, and impaired the ability of ticks to lay eggs [159]. Of significant interest, almost 20 serpins from different tick species have now been proposed for pharmacological use, since they target diverse physiological processes including blood coagulation, fibrinolysis, inflammation, and immunity [166]. Tick serpins have been shown to profoundly modulate host inflammation, and several serpins have been proposed as potential candidates for drug development against inflammatory diseases. For example, HlSerpin-a and HlSerpin-b, from the hard tick *Hae. longicornis*, displayed PI activities against multiple mammalian proteases [162]. Both suppressed the expression of inflammatory cytokines such as TNF-α, interleukin (IL)-6, and IL-1β from lipopolysaccharide-stimulated mouse bone marrow-derived macrophages (BMDMs) or mouse bone marrow-derived dendritic cells [162]. From *A. americanum*, rAAS41 inhibited chymase-mediated inflammation in rat paw edema and vascular permeability models [154]. Furthermore, some tick salivary serpins are pleiotropic, targeting both hemostatic and immune system components. Iris was the first ectoparasite serpin shown to interfere with both hemostasis and the immune response [167,168]. Iris modulated T cell and macrophage responsiveness by inducing a Th2-type response [168]. Iris also interfered with inflammation by inhibiting the production of pro-inflammatory cytokines by peripheral blood mononuclear cells [167]. Iris also was noted to bind to monocytes/macrophages and alter the secretion of TNF-α [169]. Interestingly, these activities were independent of Iris’s protease inhibitory function, making it an attractive candidate for the design of therapies for diseases with TNF-α overexpression. Finally, Iris modulates host hemostasis by targeting thrombin, FXa, and tissue plasminogen activator, thereby inhibiting platelet adhesion, blood coagulation, and fibrinolysis [168]. In contrast to Iris, the anti-inflammatory action of a second serpin from *I. ricinus*, IRS-2 [170], was solely due to its function as a serine proteinase inhibitor. IRS-2 was the first ectoparasite protein that specifically inhibited both the cathepsin G and chymase released by stimulated neutrophils and mast cells, respectively, during inflammation [170]. IRS-2 also selectively inhibited the production of IL-6 in dendritic cells stimulated with *Borrelia* spirochetes, attenuating STAT-3 phosphorylation and finally impairing Th17 differentiation and maturation [171]. Its anti-inflammatory function was confirmed in in vivo paw edema experiments, in which IRS-2 extensively inhibited edema formation and neutrophil recruitment in the inflamed tissue [170]. Moreover, this serpin inhibited cathepsin G-induced and thrombin-induced platelet aggregation, suggesting a role in hemostasis [170].

TIL domain inhibitors (trypsin inhibitor-like cysteine-rich domain) are underrepresented in tick salivary glands compared to the other protease inhibitor superfamilies but are predicted to inhibit serine proteases [12]. Several TIL domain-containing peptides were reported in the sialotranscriptomes and sialoproteomes of different tick species [98]. However, to our knowledge, only one has so far been functionally characterized: Ixodidin from *R. microplus* [172]. Ixodidin has anti-trypsin and anti-elastase properties in addition to antimicrobial activity [172].

The Kazal-type proteinase inhibitors (KPIs) were detected in tick salivary glands and are predicted to function as anticoagulants in blood-sucking animals such as leeches, mosquitoes, and ticks [98]. However, to our best knowledge, no protein from this superfamily has been functionally characterized from tick salivary glands.

Although most of the PIs found in tick saliva can be classified into the previously mentioned classes, some inhibitors defy classification. For instance, Sculptin was classified as a new thrombin inhibitor identified in a transcriptomic analysis of *A. sculptum* salivary glands. It also prolonged blood clotting times in a concentration-dependent manner [173]. This inhibitor is similar to hirudin, an important and widely-studied inhibitor from leeches, and molecules of the same class have been used clinically [174]. Sculptin was classified as a competitive, reversible, and specific thrombin inhibitor, because its inhibition mechanism was slightly different to that of hirudin; however, the agents have a similar inhibition constant (Ki). Studies with Sculptin have shown that, during inhibition, it is degraded by serinoproteases including thrombin, so it has been suggested that this inhibitor probably does not require antidotes [173]. Interestingly, Sculptin diverges phylogenetically from hirudin, and this class of inhibitors is rarely found in ticks; however, due to the importance of molecules similar to hirudin in salivary complexes from leeches, this class may warrant closer attention in ticks.

### 4.2. Tick Cysteine PIs and Their Applications

Cystatins constitute a superfamily of tight-binding inhibitors that are widely represented in various organisms and reversibly interact with papain-like cysteine proteases (family C1) and legumains (family C13). In ticks, only inhibitors of papain-like cysteine proteases have been reported to date [11], all belonging to two of four subgroups of CPIs: type 1 cystatins (stefins), which are mostly involved in intracellular blood digestion or tick developmental processes; and type 2 cystatins, the most studied in ticks, which can be secreted via their saliva to overcome host immune responses [175]. We previously reviewed the role of cystatins in tick physiology and blood feeding [175]. Since their first identification in ticks, the functions of around 20 cystatins have been experimentally validated, at least in vitro, and we list and describe these in our recent review [12], so here we only discuss those cystatins validated in vivo. Due to their immuno-pharmacological properties, tick cystatins have been proposed as therapeutics for immune-related diseases. For instance, two secreted type 2 salivary cystatins from *I. scapularis*, Sialostatin L and Sialostatin L2, have been functionally characterized and shown to have anti-inflammatory and immunosuppressive functions in vitro and in mammalian models of immune-related diseases [176,177]. Sialostatin L’s proprieties are possibly due to its inhibitory activity against lysosomal cysteine cathepsins L, C, V, S, and X and papain, which are important in matrix degradation by fibroblasts, and intracellularly for protein cleavage by antigen-presenting cells [176]. In a related study, Sialostatin L inhibited the proliferation of both CD4^+^ and CD8^+^ T cells, suggesting a modulatory effect on adaptive immunity [176,177,178]. Furthermore, Sialostatin L inhibited neutrophil migration in severe inflammation and the secretion of cytokines by mast cells, dendritic cells, and lymphocytes [176]. Sialostatin L dramatically reduced the secretion of IL-9 by Th9 cells, an essential inducer of asthma symptoms [179]. Through this inhibition, Sialostatin L abrogated airway hyperresponsiveness and eosinophilia in an experimental asthma model, probably by inhibiting IRF4 [179,180]. Another possible application of Sialostatin L was associated with its ability to decrease the production of IFNγ and IL-17 by T cells in an experimental autoimmune encephalomyelitis (EAE) mouse model of multiple sclerosis [181], in which administration of Sialostatin L significantly prevented disease symptoms [181]. Similar to Sialostatin L, Sialostatin L2 has been described as an anti-inflammatory compound since it inhibits cathepsins L, C, V, and S, with preferential affinity for cathepsins L and V [182]. Sialostatin L2 impaired inflammasome formation and inhibited caspase-1 maturation, leading to a decrease in IL-1 and IL-18 secretion by macrophages [182]. Moreover, Sialostatin L2 suppressed IFN-β-mediated immune reactions in murine dendritic cells upon infection with *Borrelia burgdorferi* [183]. DsCystatin, from the salivary glands of *Dermacentor silvarum*, interacted with human cathepsins L and B and impaired their activities [184]. DsCystatin was demonstrated to inhibit the expression of inflammatory cytokines such as IL1β, IFNγ, TNFα, and IL-6 from mouse BMDMs. DsCystatin also attenuated TLR4 signaling by targeting TRAF6 and relieved inflammation in Freund’s adjuvant-induced mouse arthritis models [184]. With a similar affinity to Sialostatins, Iristatin, a novel type 2 cystatin from *I. ricinus*, inhibited the proteolytic activity of cathepsins L and C [185]. It also reduced the production of several T cell-derived cytokines including IL-2, IL-4, IL-9, and IFN-γ, mast cell pro-inflammatory cytokines (notably IL-6 and IL-9), and nitric oxide by macrophages [185]. In addition, Iristatin inhibited CD4^+^ T cell proliferation following OVA antigen induction and hindered neutrophil and myeloid cell recruitment in vivo and in vitro. With such promising immunosuppressive activities, these cystatins may be exploitable as immunotherapeutics.

## 5. Concluding Remarks

Nature has always been a constant resource for drug discovery, providing a catalog of diverse compounds with different and interesting proprieties. Given their crucial roles in diverse physiological processes, naturally-derived PIs are major drug candidates for the treatment of several life-threatening diseases. Here, we reviewed several PIs implicated as therapies for diseases such as hypertension, AIDS, adult T cell leukemia, malaria, Alzheimer’s disease, hepatitis, and diabetes. Naturally-derived PIs have a high target specificity and selectivity and a low risk of toxicity and immunogenicity due to their low molecular weights; consequently, they are predicted to have fewer side-effects when administered at the correct doses. PIs are also seen as attractive compounds in agriculture due to their pesticide, antimicrobial, insecticide, anti-fungal, and antibacterial properties. Interestingly, some PIs from vegetable sources exhibit unique stabilities at high temperatures and extreme pH. As reviewed here, two classification schemes are often used when characterizing new PIs. Despite their functional and sometimes structural similarities, there is no unified design concept that is valid for all classes, and PI-derived drug development remains quite challenging. For efficient development of PI-derived drugs, new design concepts and technologies are required such as docking simulations, in silico screening, or in silico *de novo* design. The evolving knowledge and continuous increase in information about their structure, mechanism of action, and function pave the way for future in-depth studies.

Addressing these challenges, PIs from tick salivary glands might be regarded as “safe compounds” given their high affinity and specificity to their target protease in the host. Apart from being specific, tick PIs target several biological systems including the immune system, hemostasis, inflammation, and wound healing as well as pathophysiological processes such as tumor formation and angiogenesis. Over the last three decades, hundreds of PIs from different tick species have been characterized at the biochemical and functional levels, and some of them have been tested in advanced in vivo and preclinical trials. However, there is still no commercialized tick-derived therapy, and even the most advanced studies are still at the preclinical stage.

## Figures and Tables

**Table 1:** Examples of protease inhibitors used in agriculture and medicine.

PI	Type	Source	Application/Role	Reference
SlCYS8	Cysteine protease inhibitor	*Solanum lycopersicum*	Inhibition of herbivorous insects	[74]
PPTI	Kunitz-type inhibitor	*Poecilanthe parviflora* seeds	Pesticide	[75]
TCMP-1	Metalloprotease inhibitor	Tomato	Protect plants from cadmium accumulation	[76]
CPI	Metalloprotease inhibitor	Tomato leaves	Protect plants against herbivores attacks	[77]
PpyTI	Kunitz-type inhibitor	*Poincianella pyramidalis*	Insecticide	[78]
BWI-1	Serine protease inhibitor	Buckwheat seeds	Suppression of filamentous fungi growth	[79]
Cowpea cystatin	Cysteine protease inhibitor	Cowpea seeds	Pesticide	[80]
AtCYSa/AtCYSb	Cysteine protease inhibitor	*Arabidopsis thaliana*	Improve plants tolerance to various stresses	[81]
Maspin	Serine protease inhibitor	Humans	Tumor suppressor	[82]
MNEI	Serpin	Humans	Regulation of extravascular sites inflammation	[83]
DtTC	Serine protease inhibitor	*Derris trifoliata*	Antimalarial agent	[84]
BBI	Bowman–Birk inhibitor	Soybean	Suppresses autoimmune encephalomyelitis	[85]
BBI	Bowman–Birk inhibitor	Soybean	Anticarcinogenic agent	[86]
JPM-OEt	Cysteine protease inhibitor	-	Inhibition of tumor growth	[87]
BBI	Bowman–Birk inhibitor	Soybean, pea, lentil, and chickpea	Colorectal cancer prevention	[61]
PDI	Kunitz-type inhibitor	Potato	Trypsin and cathepsin D inhibitor	[88]
CMTI-V	Serine protease inhibitor	Pumpkin seeds	Trypsin inhibition	[89]
SPIPm2	Kazal-type inhibitor	Shrimp (*Penaeus monodon*)	Immune response against white spot syndrome virus	[90]
BILN 2061	Serine protease inhibitor	-	Antiviral effect against hepatitis C virus	[91,92]
AFLEI	Serine protease inhibitor	*Aspergillus flavus*	Elastase inhibitor	[93]

## Abbreviations

ADP	Adenosine diphosphate
BBI	Bowman–Birk inhibitor
BMDM	Bone-marrow-derived macrophages
CAM	Chicken chorioallantoic membrane
EAE	Experimental autoimmune encephalomyelitis
HIV	Human immunodeficiency virus
IFN	Interferon
IL	Interleukin
Ir-CPI	Ixodes ricinus contact phase inhibitor
IRF	IFN-regulatory factor
IrSPI	Ixodes Ricinus serine protease inhibitor
IRS-2	Ixodes ricinus serpin-2
KPIs	Kazal-type proteinase inhibitors
MMP	Matrix metallopeptidase
MPI	Metalloprotease inhibitor
NRTI	Nucleotide/nucleoside reverse transcriptase inhibitor
NNRTI	Non-nucleoside reverse transcriptase inhibitor
OVA	Ovalbumin
PBMC	Peripheral blood mononuclear cells
PDGF	Platelet-Derived Growth Factor
PI	Protease inhibitor
RCL	Reactive center loop
RENCA	Murine renal adenocarcinoma
RGD	Arginylglycylaspartic acid
RHS8	*Rhipicephalus haemaphysaloides*
ROS	Reactive oxygen species
STAT	Signal transducer and activator of transcription
TAP	Tick anticoagulant peptide
TdPI	Tick-derived protease inhibitor
TIL	Trypsin inhibitor like
TLR	Toll-like receptor
TNF	Tumor necrosis factor
TRAF	TNF receptor-associated factor
VEGF	Vascular endothelial growth factor
